# Preparation and Investigation of *Artemisia annua* L.-Loaded Alginate Hydrogels with Excipients

**DOI:** 10.3390/ph19030424

**Published:** 2026-03-05

**Authors:** Boglárka Papp, Zsolt Szűcs, Sándor Gonda, Zoltán Cziáky, Richárd Kajtár, István Lekli, Ádám Haimhoffer, Ágnes Klusóczki, Liza Józsa, Ágota Pető, Nodirali S. Normakhamatov, Zoltán Ujhelyi, Ildikó Bácskay, Pálma Fehér

**Affiliations:** 1 Department of Pharmaceutical Technology, Faculty of Pharmacy, University of Debrecen, Rex Ferenc utca 1, H-4002 Debrecen, Hungary; papp.boglarka@pharm.unideb.hu (B.P.); haimhoffer.adam@pharm.unideb.hu (Á.H.); klusoczki.agnes@pharm.unideb.hu (Á.K.); jozsa.liza@pharm.unideb.hu (L.J.); peto.agota@pharm.unideb.hu (Á.P.); bacskay.ildiko@pharm.unideb.hu (I.B.); 2 Pharmacology Doctoral Program, Doctoral School of Medical Sciences, University of Debrecen, Nagyerdei Körút 98, H-4032 Debrecen, Hungary; kajtar.richard@pharm.unideb.hu (R.K.); lekli.istvan@pharm.unideb.hu (I.L.); 3 Department of Pharmacognosy, University of Debrecen, Rex Ferenc utca 1, H-4002 Debrecen, Hungary; szucs.zsolt@pharm.unideb.hu (Z.S.); gonda.sandor@science.unideb.hu (S.G.); 4 Department of Botany, University of Debrecen, Egyetem tér 1, H-4032 Debrecen, Hungary; 5 Institute of Environmental and Natural Sciences, University of Nyíregyháza, Sóstói út 31./B, H-4400 Nyíregyháza, Hungary; 6 Ecophysiological and Environmental Toxicological Research Group, HUN-REN Balaton Limnological Research Institute, Klebelsberg Kuno utca 3, H-8237 Tihany, Hungary; 7 Agricultural and Molecular Research and Service Group, University of Nyíregyháza, Science Park, Sóstói út 31/b, H-4400 Nyíregyháza, Hungary; cziaky.zoltan@nye.hu; 8 Department of Pharmacology, Faculty of Pharmacy, University of Debrecen, Rex Ferenc utca 1, H-4002 Debrecen, Hungary; 9 Pharmaceutical Ecosystem Center, University of Debrecen, Rex Ferenc utca 1, H-4002 Debrecen, Hungary; 10 Department of Inorganic, Physical and Colloidal Chemistry, Tashkent Pharmaceutical Institute, Aybek str, 45, Tashkent 1000015, Uzbekistan; nodirali@gmail.com; 11 Department of Pharmaceutical Industry and Pharmaceutical Technology, Faculty of Pharmacy, University of Debrecen, Rex Ferenc utca 1, H-4002 Debrecen, Hungary; ujhelyi.zoltan@pharm.unideb.hu

**Keywords:** *Artemisia annua* L., hydrogel, dexpanthenol, hyaluronic acid, wound healing, casticin

## Abstract

**Background:** *Artemisia annua* L. is a medicinal plant with documented antimicrobial, antioxidant, and anti-inflammatory properties. Although widely studied for internal therapeutic applications, its topical use—especially in hydrogel-based systems—has not been thoroughly investigated. The aim of this study was to develop sodium alginate hydrogels containing *Artemisia annua* extract, supplemented with hyaluronic acid and dexpanthenol, and to evaluate their physicochemical characteristics as well as their biological activities in vitro and in vivo. **Methods:** Select bioactive constituents of the *Artemisia annua* extract were quantified using liquid chromatography coupled with electrospray ionization mass spectrometry (LC-ESI-MS). Hydrogels were prepared by cross-linking sodium alginate with a calcium carbonate–glucono-delta-lactone system and were formulated with or without hyaluronic acid and dexpanthenol. Physicochemical evaluations included measurements of moisture content, water-retention capacity, gelation time, and pH. The hydrogel microstructure was examined by scanning electron microscopy (SEM). Antioxidant activity was assessed using three methods: the 2,2-diphenyl-1-picrylhydrazyl (DPPH) assay, the ferric reducing antioxidant power (FRAP) assay, and the cupric reducing antioxidant capacity (CUPRAC) assay. Biocompatibility and regenerative effects were analyzed using cell viability assays and an in vitro scratch wound model on human keratinocyte cells. In vivo wound-healing efficacy was examined in rats with full-thickness skin excisions. **Results:** The extract contained high levels of methylated flavonoids and sesquiterpenes characteristic of *Artemisia annua*. Hydrogels supplemented with hyaluronic acid and dexpanthenol exhibited improved hydration stability and higher porosity. All formulations demonstrated measurable antioxidant activity, and those containing hyaluronic acid showed the strongest effects. The preparations were biocompatible and enhanced keratinocyte migration in vitro, with the combined hyaluronic acid–dexpanthenol formulation promoting the fastest wound closure. In vivo, *Artemisia annua* hydrogels accelerated wound healing by two to three days compared with untreated wounds. **Conclusions**: These results confirm the promise of *Artemisia annua* hydrogels for topical wound care and highlight the beneficial contributions of hyaluronic acid and dexpanthenol to their structural and therapeutic performance.

## 1. Introduction

*Artemisia annua* L. (AA) is a medicinal plant that belongs to the Asteraceae family, commonly known as sweet wormwood or Qinghao [1]. It is an annual herb native to Asia, now distributed in Europe, Africa, and North America. It has been used in traditional Chinese medicine for thousands of years for the treatment of fever and infectious diseases such as malaria [2]. The medicinal value of AA extends beyond malaria, as it has been explored for its antiviral, antifungal, antimicrobial, anticancer, and anti-inflammatory properties [1,3]. AA has shown potential antidiabetic effects by improving glucose metabolism and enhancing insulin sensitivity by reducing the glucose levels and increasing insulin levels in the sera of diabetic rats [4,5]. A wide range of phytochemicals, including sesquiterpenoids, flavonoids, coumarins, lipids, phenolics, purines, steroids, triterpenoids, aliphatic substances, and the primary bioactive compound artemisinin, have been described from AA [6,7].

There is an extensive body of evidence available on the internal use of the herb; however, there are few studies on the topical use of AA and its potential excipients. Huang et al. demonstrated the positive effect of the drug on atopic dermatitis in mice with a topical formulation of AA essential oil [8]. Bao et al. investigated the efficacy of artemisinin-loaded hydrogels in rats to promote wound healing and the anti-tumor effects of the formulations [9].

Hydrogels are three-dimensional, hydrophilic polymer networks that can retain large amounts of water, making them highly useful for biomedical applications such as wound healing, drug delivery, and tissue engineering [10,11]. Their biocompatibility, ability to mimic natural tissue environments, and capacity to control the release of therapeutic agents contribute to their widespread use in the medical and pharmaceutical fields.

Sodium alginate, a naturally derived polysaccharide from brown seaweeds, is widely used in the food, pharmaceutical, and biomedical industries for its biocompatibility and non-toxic properties [12,13]. It is characterized by its ability to form gels in the presence of divalent cations like calcium, which cross-link its polymer chains to create a stable gel structure [14]. In hydrogels, sodium alginate plays a key role by providing a structure for controlled drug release, cell encapsulation, and wound healing, due to its ability to absorb water and maintain a moist environment [15,16].

Hydrogels are considered highly effective for topical drug delivery due to their high water content, biocompatibility, and versatility in encapsulating various drug types [11]. Recent studies also highlight that hydrogel formulations can be engineered with specific polymer networks and active extracts to enhance antioxidant, antibacterial, and cell-proliferative properties, further supporting their therapeutic potential in skin applications like wound healing [17,18]. Their water-rich structure enables them to maintain a moist environment on the skin, which enhances drug absorption and aids in wound healing [19,20]. Hydrogels can also be modified to control drug release rates, allowing for sustained and localized drug delivery over extended periods [21]. Additionally, their three-dimensional polymeric network provides structural stability while facilitating efficient interaction between the drug and the skin surface [22].

Hyaluronic acid (HA), often used in topical formulations at concentrations ranging from 0.1% to 2%, provides powerful hydration by binding moisture to the skin, enhancing skin elasticity, and reducing the appearance of wrinkles [23]. In addition to its hydrating properties, HA also acts as a skin barrier enhancer, decreasing transepidermal water loss (TEWL) and protecting against environmental stressors [24].

Dexpanthenol (DP), a derivative of pantothenic acid (vitamin B5), is a biologically active compound that penetrates the skin efficiently, where it is converted to pantothenic acid, promoting wound healing, skin hydration, and cellular regeneration [25]. The concentration of dexpanthenol in topical formulations typically ranges from 2% to 5%, providing optimal skin penetration and enhancing wound healing and hydration effects. Proksch et al. showed that dexpanthenol-containing formulations can accelerate wound healing and reduce the time to wound closure, which is crucial for minimizing transepidermal water loss (TEWL) and lowering the risk of infection [26].

Transdermal drug delivery is a critical advancement in pharmaceutical science, enabling the controlled and sustained release of therapeutics through the skin directly into systemic circulation, thereby enhancing bioavailability, minimizing first-pass metabolism, and reducing adverse effects [27]. Matrix systems embedded in natural polymers offer biocompatible, biodegradable, and sustained drug release while minimizing skin irritation, simplifying application, and enhancing tissue integration—making them ideal for safe and effective drug delivery [28].

The aim of this study was to formulate a stable and well-tolerated drug carrier for AA extracts with different excipients, and to evaluate the anti-inflammatory, antioxidant, and wound-healing effects of these formulations. Hydrogels are potentially good drug carriers for external drug delivery, and the safety of their internal use of sodium alginate has been mentioned in a previous article [29]. In order to characterize the formulations, different dosage form studies were performed, and the moisture content, water retention, pH, and gelation time of the hydrogels were measured. The quantification of select bioactive constituents of AA was performed using LC-ESI-MS. In vitro biocompatibility, cytotoxicity, wound-healing, and anti-inflammatory activity assays were performed on human keratinocyte cells (HaCaT). DPPH, FRAP, and CUPRAC antioxidant assays were performed to investigate the antioxidant effects of the formulations. The in vivo anti-inflammatory effects of the formulations were investigated in a rat paw edema model. The rats were incised and monitored continuously for two weeks to investigate the wound healing-promoting effects of the selected preparations.

## 2. Results

### 2.1. Quantification of Key Bioactives of the Artemisia annua Extract

The dry extract contained 108.36 ± 5.28 ng/mg casticin and 15.83 ± 2.21 ng/mg artemetin. In addition, sesquiterpenoids characteristic of *A. annua* were quantified, including traces of artemisinin, 2.30 ± 0.06 ng/mg arteannuin B, 24.92 ± 0.41 ng/mg deoxyartemisinin, and 11.03 ± 0.94 ng/mg dihydroartemisinic acid. All compound concentrations are expressed as ng/mg dry extract.

The determination of the bioactive compounds was found from an LLOQ of 0.01–0.5, depending on the metabolite, up to a ULOQ of 20 μg/mL in positive ion mode for the tested flavonoids and sesquiterpenes. The calibration curve performance indicators and coverage are shown in Table S1. Extracted ion chromatograms are available (Figures S1–S6).

### 2.2. Results of Gelation Time, Water-Retaining Capacity, Moisture Content, and pH

The results showed that the formulations containing DP (VII.–VIII.) had better water- and moisture-retention properties than the formulations without excipients or containing only HA. The pH values were around pH 5 for all four formulations, with the sample containing HA showing the lowest pH value. The average pH of the skin is 5.0–6.0, resulting in a higher penetration of topical products with a similar pH. The results are presented in Table 1 [30].

### 2.3. Scanning Electron Microscopy (SEM)

**Figure 1:**
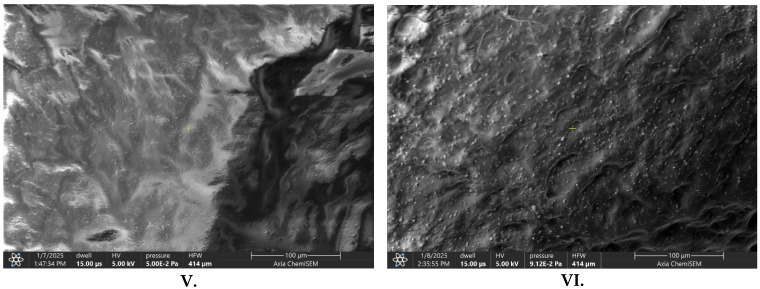

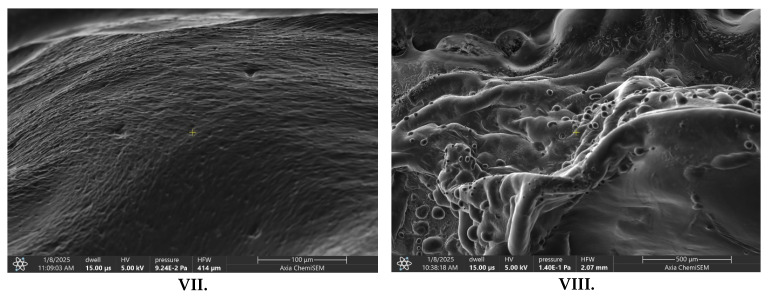
Scanning electron microscope images of different AA-containing hydrogels without excipients (**V**.), with hyaluronic acid (**VI.**), with dexpanthenol (**VII**.), and with both excipients (**VIII**.).

SEM analysis demonstrated significant alterations in the surface morphology of AA-loaded hydrogels, depending on excipient composition. The SEM images are shown in Figure 1. The formulation without excipients (V.) exhibited a compact, smooth surface with minimal porosity. Addition of HA (VI.) led to the formation of a porous, sponge-like structure, suggesting increased hydrophilicity and potential for enhanced fluid exchange [31]. The DP-containing hydrogel (VII.) showed a fibrous and stratified architecture, indicative of modified internal organization. The combination of HA and DP (VIII.) resulted in a highly porous, heterogeneous network, which may promote improved drug release kinetics and cellular interactions relevant to wound-healing applications [32]. These observations are based on qualitative visual inspection of SEM images, and no quantitative morphometric analysis of pore size or porosity was performed

### 2.4. Results of Synthetic Membrane Diffusion Model of Casticin

**Figure 2:**
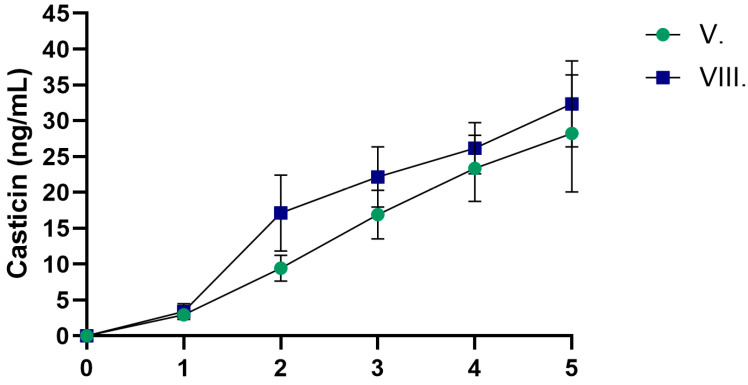
Dissolution profiles of casticin from formulations V and VIII. Formulation V. contained *Artemisia annua* (AA) extract, while formulation VIII was composed of AA, hyaluronic acid (HA), and dexpanthenol (DP). Data are presented as the mean ± SD (*n* = 6).

Figure 2 presents the dissolution profiles of casticin from the two tested formulations. Formulation V contained *Artemisia annua* (AA) extract alone, whereas formulation VIII combined AA with hyaluronic acid (HA) and dexpanthenol (DP). Both formulations showed a time-dependent increase in casticin release, indicating sustained release of the compound. Overall, formulations VIII and V were not significantly different in terms of casticin release. It should be emphasized that the synthetic membrane model used in this study represents a simplified diffusion system and does not reproduce the structural and functional barrier properties of human skin. Therefore, the obtained data reflect only the relative in vitro release behavior of the formulations and cannot be used to predict dermal penetration, transdermal delivery, or in vivo bioavailability.

### 2.5. Results of Antioxidant Property Measurements

#### 2.5.1. FRAP (Ferric Reducing Antioxidant Power)

**Figure 3:**
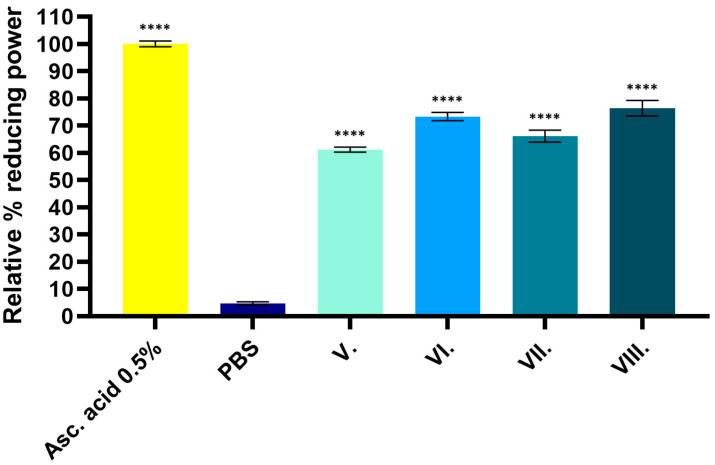
Relative percentage of reducing power determined by the FRAP assay. Ascorbic acid (5 mg/mL%) was used as a positive control and as 100%. PBS was used as the negative control, and the reducing power of the formulations (samples V–VIII) was evaluated relative to it. Data are expressed as the mean ± SD (*n* = 6). Statistical significance is indicated as **** *p* < 0.0001.

Figure 3 shows the FRAP of the tested samples expressed as a percentage of ascorbic acid. The results demonstrate that gels containing AA have significant antioxidant properties (V–VIII). Higher antioxidant activity can be observed for samples containing HA (formulations VI. and VIII.) than for samples without excipients or containing only DP. The statistical analysis of the results is presented in Table 2.

#### 2.5.2. DPPH (2,2-diphenyl-1-picrylhydrazyl Antioxidant Assay)

**Figure 4:**
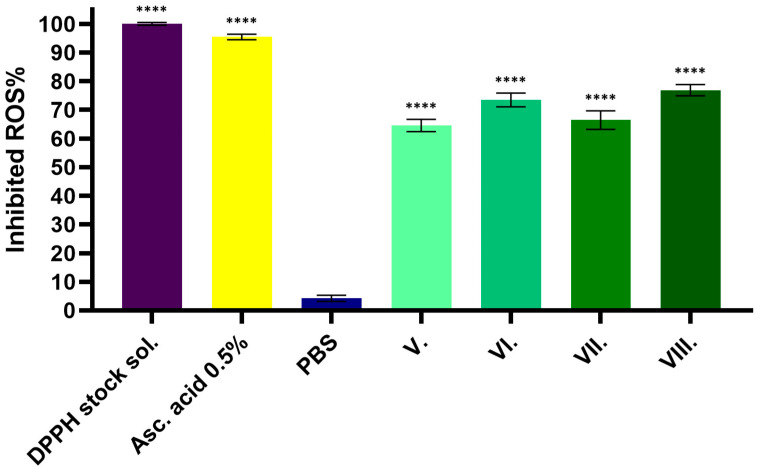
Percentage of inhibited reactive oxygen species (ROS%) measured using the DPPH assay: 5 mg/mL% ascorbic acid was used as a positive control, while PBS served as a negative control. All test samples (V.–VIII.) showed significantly higher activity compared to the negative control (PBS) (**** *p* < 0.0001). Data are expressed as the mean ± SD (*n* = 6).

The radical-scavenging activity of the four formulations was expressed as a percentage (SA%) of the DPPH test solution. Ascorbic acid (5 mg/mL%) with strong radical-scavenging behavior was used as a positive control and PBS as a negative control, which was the solvent for the test solutions. The results showed that the samples containing both excipients (formulation VIII.) presented the highest radical-scavenging activity. Therefore, AA with hyaluronic acid showed higher radical-scavenging activity than the herb formulation alone (formulation V.). The results are shown in Figure 4. The statistical analysis of the results is presented in Table 3.

#### 2.5.3. CUPRAC (Cupric Reducing Antioxidant Capacity)

**Figure 5:**
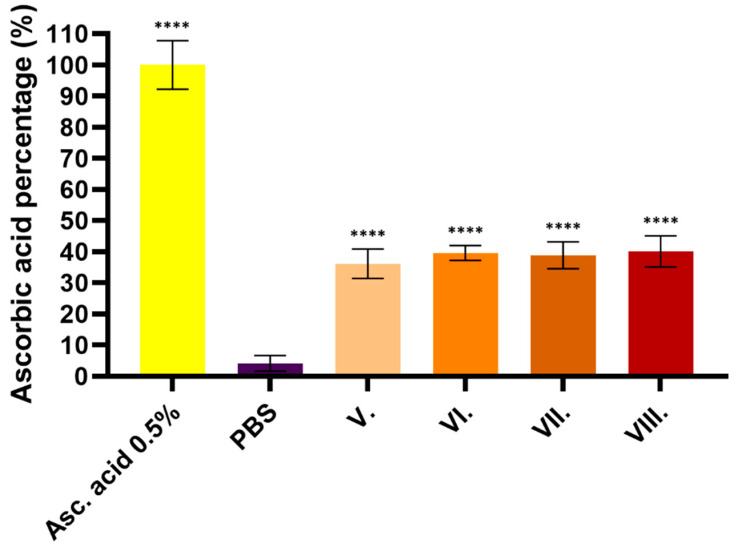
Antioxidant capacity was evaluated using the CUPRAC assay. Results are presented as a percentage relative to the negative control (PBS), while 5 mg/mL ascorbic acid was included as a positive control. Data are expressed as the mean ± SD (*n* = 6). Statistical significance is indicated as **** *p* < 0.0001.

The CUPRAC method is based on the reduction of copper (II) ions, during which antioxidant molecules transfer electrons to copper (II) ions, converting them into copper (I) ions. The absorbance of the resulting colored copper (I)–neocuproine complex can be measured spectrophotometrically; its concentration is considered proportional to the antioxidant capacity. The results are shown in Figure 5. For all four formulations, antioxidant properties above 30% were measured relative to ascorbic acid. However, no significant difference was detected between the formulations. The statistical analysis of the results is presented in Table 4.

### 2.6. MTT Cytotoxicity Assay

**Figure 6:**
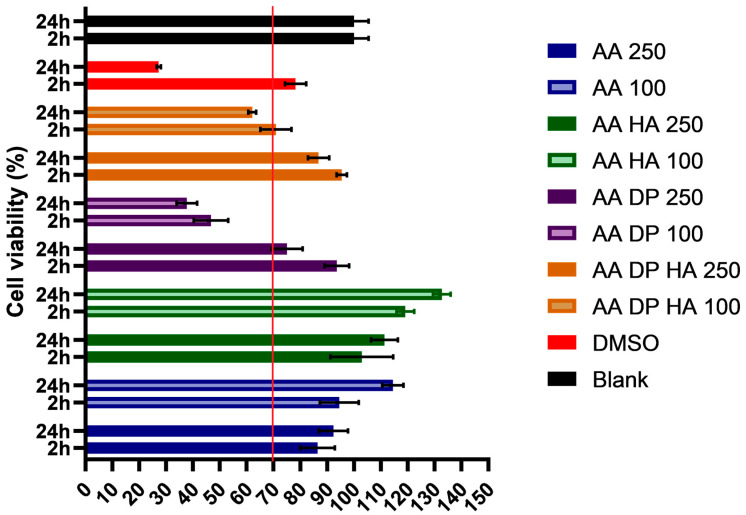
Cell viability test with the MTT assay on HaCaT cells after incubation with the formulations for 2 h and 24 h. Cell viability is expressed as the percentage of the negative control (PBS); 0.50 *V*/*V*% DMSO served as the positive control. Each data point represents the mean ± SD, and *n* = 6. Formulation V. is represented by AA 250 and AA 100, corresponding to two different concentrations. Formulation VI is denoted as AA HA, formulation VII as AA DP, and formulation VIII as AA HA DP. The red line in the figure indicates viability reaching 70%.

In this study, the AA extract was evaluated at concentrations of 100 µg/mL and 250 µg/mL in combination with various excipients to assess its effects on HaCaT cell viability using the MTT assay. Formulations containing only hyaluronic acid (AA + HA 100 µg/mL and AA + HA 250 µg/mL) exhibited the highest viability, exceeding 120%, indicating enhanced cellular metabolic activity and biocompatibility. In contrast, AA samples containing only dexpanthenol showed reduced viability, particularly at 100 µg/mL, but remained above the 70% cytotoxicity threshold defined by ISO 10993-5:2009 [33]. The DMSO positive control showed significantly reduced viability, confirming assay sensitivity and validating the safety profile of the experimental samples. The results are shown in Figure 6. The statistical analysis of the results is presented in Table 5.

### 2.7. Results of In Vitro and In Vivo Wound-Healing Effects of AA-Loaded Hydrogels

**Figure 7:**
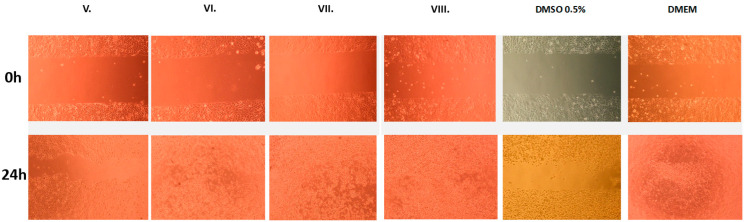
Images of the closure rate after removal of the silicone insert at hour 0 and at hour 24. A 0.5% DMSO solution was used as a positive control, and a DMEM solution was used as an untreated control. The initial cell-free area measured at hour 0 was 500 μm in all cases after removal of the silicone septum.

The in vitro wound-healing assay revealed differences in wound closure among the treatment groups. The DMEM control group exhibited excellent wound closure, indicating strong baseline cell migration and proliferation. Among the treated samples, the VIII. group showed the most pronounced enhancement in wound healing, with nearly complete closure of the scratch area. Formulations V., VI., and VII. also promoted considerable cell migration, albeit to a slightly lesser extent. In contrast, the DMSO 0.5% group showed poor cell migration, suggesting a possible inhibitory effect. These results suggest that the AA-based formulations, particularly those combined with DP and HA, are associated with enhanced wound-closure behavior in vitro. The images of the wound closure are shown in Figure 7.

**Figure 8:**
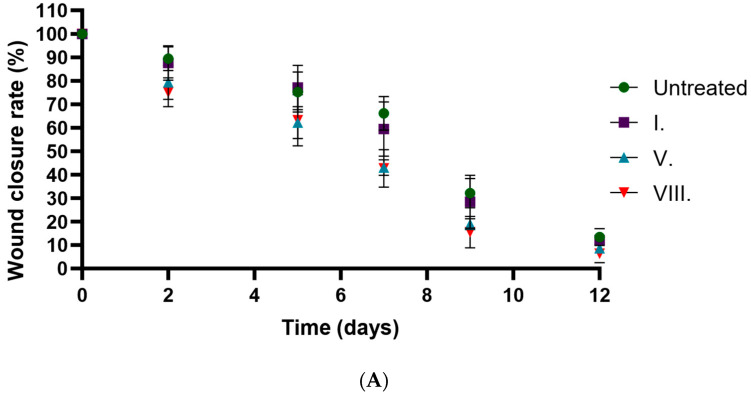

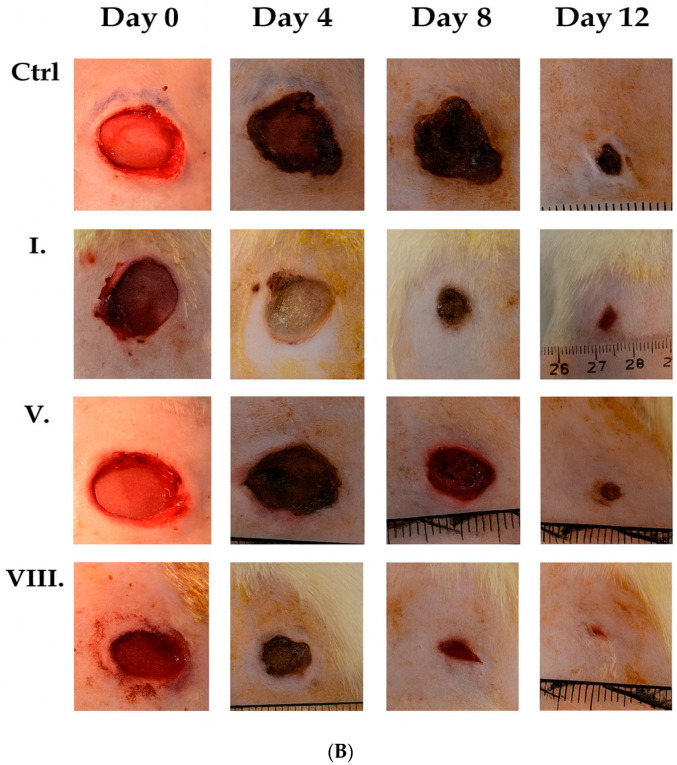
(**A**) Time-course plot of wound-closure rate (%) versus days post-injury in an in vivo wound-healing model. (**B**) Representative macroscopic images of excisional wound healing in vivo. Full-thickness excision wounds were treated with hydrogels containing AA extract, AA combined with hyaluronic acid and dexpanthenol, or control formulations (empty hydrogel or untreated). Serial photographs of the wound sites were captured at designated time points (days 0, 2, 5, 7, 9, and 12 post-injury) to monitor wound closure and tissue regeneration over time.

The in vivo wound-closure results are presented in Figure 8. The statistical analysis of the results is presented in Table 6. Formulations containing AA were associated with faster wound closure, with closure occurring 2–3 days earlier compared to the untreated group and the empty hydrogel group. No marked difference was observed between formulations V. and VIII. Compared to the formulation without an active ingredient (formulation I.), AA-containing formulations showed smaller wound areas at the examined time points. As two wounds were generated per animal, the in vivo data include within-animal dependence, and statistical comparisons should therefore be interpreted cautiously. Overall, these findings provide supportive evidence for improved wound healing in AA-containing formulations.

## 3. Discussion

The bioactive compounds of *Artemisia annua* have attracted considerable interest due to their therapeutic potential, particularly in topical formulations [34,35]. This study aimed to develop a novel topical dosage form that optimizes the release and biological activity of these active constituents. Limited data are available on the topical use of *Artemisia annua*. Huang et al. investigated topically applied oils containing AA, and Jiu et al., in their study of cosmetics containing AA, discovered the beneficial effects of the herb in the treatment of certain skin disorders [8,36,37]. Han et al. showed that topical application of AA water extract significantly attenuated DNCB-induced atopic dermatitis in BALB/c mice by suppressing Th2 cell-mediated inflammatory responses, suggesting its potential as an effective treatment for inflammatory skin conditions [38]. However, no further evidence was identified concerning the topical application of *Artemisia annua* beyond the studies mentioned. This gap highlights the need for more systematic evaluations of AA-based topical delivery systems, particularly those capable of controlled release and prolonged skin interaction. In particular, data are lacking on the incorporation of its bioactive constituents into hydrogel-based delivery systems and the subsequent evaluation of their therapeutic performance.

In this study, different sodium alginate hydrogels containing *Artemisia annua* L. extract were prepared with different excipients. The hydrogels were prepared by ionically cross-linked gelation [39,40,41] of AA extract dissolved in a sodium alginate polymer solution with a CaCO_3_–GDL complex, which was responsible for the cross-linking in the final mixture. Dexpanthenol, which has skin-protective, moisturizing, and healing properties, was used as an excipient [42,43,44]. It is a precursor of pantothenic acid (vitamin B5), which is essential for maintaining healthy skin [25]. Clinical studies have demonstrated that 5% dexpanthenol promotes faster skin regeneration, reduces transepidermal water loss, and enhances hydration, making it effective for treating various skin conditions [45]. Gorski et al. demonstrated that the topical use of 5% dexpanthenol water-in-oil formulations significantly accelerates skin barrier restoration and wound healing on freshly tattooed skin by reducing transepidermal water loss and promoting skin hydration, protection, and regeneration when applied 4–8 times daily for 14 days [42].

Hyaluronic acid (HA) is widely used in topical formulations because of its exceptional ability to retain moisture and provide hydration. HA supports skin healing and repair by maintaining a moist environment that promotes tissue regeneration, making it an important base for wound-healing processes [46,47]. Xiaojuan Li et al. demonstrated that hyaluronic acid–poloxamer (HA-POL) hydrogel significantly promotes skin wound healing by enhancing fibroblast accumulation, granulation tissue formation, angiogenesis, and collagen deposition while providing better air permeability and antibacterial protection compared to traditional wound coverings [48]. H. Ying et al. showed that collagen/hyaluronic acid (COL–HA) hydrogels enhance wound repair by promoting the proliferation of endothelial cells and fibroblasts, increasing VEGF expression, and supporting vascular regeneration, leading to improved healing outcomes in full-thickness wounds compared to commercial treatments [49,50]. These findings underline the versatility of HA as a multifunctional excipient that can be tailored to improve both mechanical and biological properties of hydrogel systems.

In order to determine the physicochemical parameters of the hydrogels, different pharmaceutical form studies were conducted. The moisture content of the hydrogels is an important parameter in the stability studies [51]. The water-retention capacity and moisture content of the hydrogels were measured. The water content of the hydrogels remained above 60% for all formulations even after 3 days of standing. At high temperatures (120 °C), the moisture content evaporation was above 80% in all cases. Lower water loss was measured for samples containing dexpanthenol in both experiments, suggesting the contribution of dexpanthenol to the water-retention parameter of the final product [52]. Improved hydration stability is particularly advantageous for wound dressings, as maintaining a moist environment accelerates epithelialization and reduces scarring.

The use of hydrogels is common in tissue engineering or in the formulation of drug delivery systems [53]. Therefore, the pH must be within a range compatible with the human body, with the skin’s surface pH averaging 5.0–6.0 [54]. Extreme pH values could cause irritation or tissue damage [55]. The determination of the gel’s settling time is an important parameter for assessing homogeneity, as excessively fast gel settling leads to heterogeneous drug distribution and morphologically different gels [56,57].

The SEM analysis revealed that hydrogels incorporating hyaluronic acid and dexpanthenol exhibited enhanced porosity and structural heterogeneity, which are critical factors for effective wound-healing applications. Increased porosity facilitates improved cell infiltration and nutrient diffusion, essential for tissue regeneration. Choi et al. demonstrated that macroporous hydrogel structures significantly promote fibroblast proliferation and migration, underscoring the importance of scaffold architecture in tissue engineering [58]. Our results are consistent with these findings, indicating that the inclusion of HA and DP likely modified the internal network architecture, contributing to improved biological responses. A limitation of the present study is that SEM analysis was restricted to qualitative morphological assessment. Quantitative evaluation of pore size distribution and porosity was not performed. Therefore, structural differences between formulations should be interpreted as descriptive rather than statistically validated.

Cell viability studies of the formulations are crucial for the evaluation of cytotoxicity. In order to determine biocompatibility, an MTT assay was performed on the HaCaT cell line [59]. The elevated viability in VI. hydrogels suggests that HA enhances the regenerative capacity of the formulation, in agreement with reports that HA supports keratinocyte proliferation and wound closure [60].

Although dexpanthenol-only formulations sometimes showed reduced metabolic activity in vitro, treated groups often remained non-cytotoxic according to biomedical standards (e.g., viability thresholds similar to those in ISO 10993-5 [33]). For instance, topical dexpanthenol has been shown in a review to accelerate wound healing and enhance re-epithelialization and restoration of skin barrier function after superficial skin injury or cosmetic procedures [42]. In parallel, biomaterials research with hyaluronic acid (HA) supports its potential in wound healing: HA-based hydrogels—including injectable or self-healing hydrogels—have been reported to reduce inflammation, regulate tissue remodeling, and support tissue regeneration in skin wounds [61]. In effect, combining a moisturizing/barrier-restoring agent like dexpanthenol with a structural, ECM-mimicking scaffold such as an HA hydrogel could offer additive (or even synergistic) benefits, ensuring a moist, biocompatible environment while providing structural support and promoting re-epithelialization and tissue regeneration [49,62]. These concentration-dependent effects underscore the importance of optimizing the AA dosage within hydrogels to maximize therapeutic benefit while minimizing cytotoxicity.

To measure the radical-scavenging activity of the formulations, three colorimetric methods were performed. The FRAP, CUPRAC, and DPPH assays are widely used for assessing antioxidant activity, with FRAP measuring the ability of antioxidants to reduce ferric ions (Fe^3+^ to Fe^2+^) and DPPH evaluating the scavenging capacity of antioxidants against free radical species, both serving as indicators of a compound’s potential to neutralize oxidative stress [63,64,65]. HA improved the radical-scavenging activity of AA in both formulations VI. and VIII. The radical-scavenging activity of hyaluronic acid is based on electron/hydrogen donation from its free carboxyl group [66,67]. On the other hand, no significant antioxidant properties have been measured for DP based on previous studies [68]. The antioxidant activity was not adversely affected by the matrix composition, indicating that no chemical incompatibility with the plant-derived antioxidants occurred during formulation. The higher activity observed was most likely attributable to chemical interactions between the phytocomponents and DP. This suggests that complexation or microenvironmental changes within the hydrogel matrix may modify antioxidant profiles, an aspect that warrants further mechanistic investigation. The FRAP, DPPH, and CUPRAC assays are chemical in vitro methods that reflect antioxidant capacity under simplified conditions and do not directly indicate biological activity or wound-healing mechanisms. Therefore, these results should be interpreted as relative chemical indicators rather than evidence of biological effects.

AA-based hydrogels significantly improved both in vitro and in vivo wound healing, with formulation VIII. showing the strongest enhancement, likely due to the combined pro-migratory and regenerative effects of HA and DP [52,69]. HA is known to promote fibroblast migration, angiogenesis, and moisture retention, which aligns with the accelerated wound closure observed in HA-containing formulations [70]. Furthermore, hyaluronic acid has been shown to enhance biocompatibility and support cellular activities. Yang et al. reported that hyaluronic acid-based hydrogels effectively promote wound healing by supporting cell proliferation and migration [71]. DP further supports epithelialization and barrier repair, contributing to the improved healing dynamics seen in treated groups. DP can improve skin permeability by disturbing the packing in the interfacial headgroup layer of the lipid layer [42]. Low concentrations of DMSO (~5 mM) enhance fibroblast proliferation and wound healing, whereas higher concentrations (≥20 mM) exert inhibitory effects on cell growth [72]. In the in vitro scratch assay, wound closure reflects the combined effects of cell migration and proliferation. As proliferation inhibitors such as mitomycin C were not applied in the present study, the relative contribution of these two processes cannot be distinguished. Therefore, the observed effects should be interpreted as overall wound closure rather than as a direct measure of cell migration. Future studies will incorporate proliferation-blocking conditions to clarify the specific mechanisms involved. AA may contribute substantially to the observed biological effects, based on its documented anti-inflammatory and antioxidant properties; however, its individual contribution cannot be fully separated from those of the excipients in the present study [73]. The combination of AA with HA and DP may provide additive or potentially synergistic effects; however, this hypothesis requires confirmation in future studies employing appropriate factorial control designs [74]. These observations suggest the possibility of additive or synergistic interactions; however, definitive conclusions cannot be drawn without systematic excipient-only and factorial controls. It should be noted that hyaluronic acid and dexpanthenol are well-established bioactive excipients with documented wound-healing and skin-regenerative properties. As excipient-only control formulations were not systematically included in all biological assays, the individual contributions of AA, HA, and DP cannot be fully distinguished in the present study. Therefore, the observed biological effects are likely the result of combined and potentially synergistic interactions among these components, rather than being attributable solely to the plant extract. A limitation of the present in vivo study is that two wounds were generated per animal, which may introduce within-subject dependence. Although wounds were assigned to different treatment conditions in a paired design, statistical analyses were conducted primarily at the wound level and did not explicitly model animal-level random effects [75]. As systematic excipient-only and full factorial control formulations were not included in all biological assays, the present study does not allow definitive discrimination between additive and synergistic effects. Therefore, potential synergy should be regarded as a hypothesis requiring further experimental validation. The in vivo findings should be regarded as supportive evidence of improved wound closure rather than as definitive proof based solely on statistical significance. Another limitation of the present in vivo study is that wound healing was evaluated primarily by macroscopic wound area measurements without histological or immunohistochemical analysis. Therefore, the underlying tissue-level mechanisms could not be fully characterized. Future studies should apply mixed-effects models or animal-level analyses to further strengthen statistical inference.

## 4. Materials and Methods

### 4.1. Materials

A commercial dried extract of *Artemisia annua* L. (drug-to-extract ratio 1:30), manufactured using a water/ethanol solvent system, was obtained from Natürlich lang leben UG© (Coburg, Germany; batch number: 73162). According to the certificate of analysis provided by the manufacturer, the extract was produced using a water/ethanol solvent system and subsequently dried to obtain the final powder. Low-viscosity-grade sodium alginate was obtained from BÜCHI Labortechnik AG (Flawil, Switzerland). D-(+)-glucono-δ-lactone (CAS No.: 90-80-2), D-Panthenol (CAS No.: 81-13-0), hyaluronic acid sodium salt from *Streptococcus equi* (CAS No.: 9067-32-7), calcium carbonate (CAS No.: 471-34-1), and FRAP assay kits (P. No.:MAK509) were purchased from Sigma-Aldrich (St. Louis, MI, USA). The MTT dye 2-4,5-dimethyl-2-thiazolyl)-3,5-diphenyl-2H-tetrazolium bromide, Dulbecco’s Modified Eagle’s Medium (DMEM), phosphate-buffered saline (PBS), trypsin from porcine pancreas, ethylene-diamine-tetra-acetic acid (EDTA), heat-inactivated fetal bovine serum (FBS), L-glutamine, 2,2-diphenyl-1-picrylhydrazyl (DPPH), 96% *V*/*V*% ethanol and (±)-6-hydroxy-2,5,7,8-tetramethylchromane-2-carboxylic acid (Trolox) (CAS Number: 53188-07-1), L-ascorbic acid (CAS No.: 50-81-7), neocuproine (CAS No.: 484-11-7), copper (II) chloride dihydrate (CAS No.: 10125-13-0), and ammonium acetate (CAS No.: 631-61-8) were purchased from Sigma-Aldrich (Budapest, Hungary). Non-essential amino acid solution and penicillin–streptomycin mix, 12-well plates, 24-well plates, 96-well plates, and cell culture flasks were obtained from Thermo-Fisher (Darmstadt, Germany, CAS number: 156499). HaCaT cell lines (human keratinocyte cells) were obtained from Cell Lines Service (CLS, Heidelberg, Germany).

### 4.2. Quantification of Key Bioactives in the Artemisia annua Extract

To quantify the bioactive natural products from the dried AA extract, a 100 mg aliquot of the dry preparation was extracted with 1 mL of 96% EtOH to ensure efficient solubilization of the target phytochemicals prior to LC–ESI–MS analysis. The sample was shaken on a Vortex Genie for 5 min, followed by sonication for 5 min. After centrifugation and filtration, 100–1000 µg dry weight equivalent (1–10 µL) was directly injected for quantification of bioactives, along with an at least five-point calibration curve for casticin, artemisinin, dihydroartemisinic acid, arteannuin, artemetin, and deoxyartemisinin. The reference standard constituents were purchased from Sigma-Aldrich. Chromatographic parameters are described in Section 4.8.2.

### 4.3. Formulation of Artemisia annua-Loaded Alginate Hydrogels

The alginate-based hydrogels were prepared according to the method of Catazano et al. [76]. Initially, an alginate solution (10 mg/mL%) was prepared by swelling low-viscosity sodium alginate (SA) in distilled water overnight, protected from light at RT with continuous magnetic stirring. To the final alginate polymer solution, solid calcium carbonate (CaCO_3_) was added with continuous stirring to give an SA-CaCO_3_ suspension (30 mM). To the suspension, the final D-(+)-glucono-δ-lactone (GDL) solution (64 mM) was added with continuous magnetic stirring at 300 rpm. To some of the prepared suspensions, D-Panthenol (DP) (5 *V*/*V*%) was added, and finally 20% hyaluronic acid (HA) was also mixed in (20% relative to the mass of alginate). Finally, *Artemisia annua* (AA) dry extract was added to the suspension (5 mg/mL), and 0.5 mL of solution was pipetted into a 24-well plate. The compositions of the different formulations are presented in Table 7. The plate was covered with parafilm, and the formulations were kept at +4 °C for 24 h, away from light, to gel. After the gelation process, the gels were kept at +4 °C [57,76].

### 4.4. Water-Retention Capacity (WRR%)

To determine water retention, the weight of the finished hydrogels was measured, and the hydrogels were left to stand at RT with humidity of 40–50% for 3 days. The weight of the formulations was measured again, and the water-retention capacity was determined in relation to the initial weight according to the following equation:

(1) $$WRR(\%)=\frac{W_{3}}{W_{0}}\times 100$$

where W_0_ indicates the initial mass of the formulation, while W_3_ indicates the mass of the formulation after three days [77].

### 4.5. Moisture Content

To measure the moisture content of the hydrogels, a KERN DAB moisture analyzer (Albstadt, Germany) was used. This instrument measures the initial weight, and then at 120 °C it continuously measures the change in weight due to evaporating water content, expressed as a percentage [78].

### 4.6. Determination of Gelation Time and pH

To determine the time to gelation, the formulations were poured into a 24-well plate after mixing and then left to solidify at RT. The gelling time of hydrogels was measured after the addition of GDL by manual verification of GDL-CaCO_3_ complex formation. The endpoint of the gelation was when the samples had solidified on the plate, as assessed by the lack of flow during the 45° rotation of the plate [79].

The pH of the formulations was measured using the FiveEasy Plus pH meter FP20 (Mettler Toledo, Greifensee, Switzerland). The formulations were pre-cast on a 6-hole plate with a minimum thickness of 1 cm to perform the measurement.

### 4.7. Scanning Microscopy Pictures of Hydrogels

The morphological characteristics of sodium alginate-based hydrogels incorporating various excipients were investigated using a Thermo Scientific™ Axia™ ChemiSEM™ Scanning Electron Microscope (Auro-Science Consulting, Budapest, Hungary). Hydrogel samples were affixed to aluminum stubs using double-sided adhesive carbon tape. Excess non-adherent material was removed by using a gentle argon gas flow to minimize surface contamination. No surface coating, pre-treatment, or post-acquisition image processing was applied. Scanning was performed under high-vacuum conditions with an accelerating voltage of 20 kV and a dwell time of 15 µs. All micrographs were acquired at a fixed magnification of 10,000×.

### 4.8. Synthetic Membrane Diffusion Model on Franz Diffusion Cell Apparatus

To determine the in vitro drug release of the hydrogels, a Franz diffusion cell (Hanson MicroetteTM Topical and Transdermal Diffusion Cell System) was used for measurement. A synthetic cellulose acetate membrane was employed as a simplified diffusion barrier for comparative release studies, rather than as a physiological model of human skin. The membrane was inserted between the donor and acceptor phases, and sample aliquots of 1000 µL were collected at defined intervals (0, 60, 120, 180, 240, and 300 min). The membrane diffusion area was 1.767 cm^2^, the membrane pore diameter was 0.45 µm, and freshly prepared 30% ethyl alcohol (7 mL/cell) was used as the acceptor phase. To achieve homogeneous drug distribution and uniform contact between the donor phase and the membrane, the medium was kept under continuous magnetic stirring (300 rpm) and at 32 ± 0.5 °C. From each composition, 0.5 mL of each formulation was placed on the membrane, corresponding to 2.5 mg of AA in the preparation. Casticin was selected as the marker compound for diffusion studies because it is a major flavonoid constituent of AA extract, can be reliably quantified by LC–ESI–MS, and is biologically relevant due to its antioxidant, anti-inflammatory, and wound-healing-related properties [80,81,82]. This experimental setup was used to compare the relative in vitro release profiles of casticin from different formulations, and it does not provide information on skin permeation or in vivo bioavailability.

#### 4.8.1. Sample Preparation

Acceptor phase aliquots of 1000 μL in volume were obtained and stored at −80 °C until further processing. Aliquots were evaporated to dryness in an RVC 2-33 CDplus rotary vacuum evaporator (Martin Christ, Osterode am Harz, Germany) and redissolved in 500 μL of 96% ethanol. Five-point calibration curves from casticin (Sigma-Aldrich) were constructed as 0.001, 0.01, 0.05, 0.1, and 0.5 μg/mL with 96% ethanol and used as external calibration (R^2^ = 0.9994).

#### 4.8.2. Mass Spectrometry

A UHPLC system (Dionex Ultimate 3000RS) was coupled with a Thermo Q Exactive Orbitrap mass spectrometer (Thermo Fisher Scientific Inc., Waltham, MA, USA) equipped with an electrospray ionization source (ESI). The HPLC separation was achieved on a Phenomenex Kinetex XB-C_18_ column (100 mm × 2.1 mm × 2.6 μm). Oven temperature was maintained at 30 °C, and the flow rate was 250 μL/min. Eluent A was water containing 0.1% formic acid, and eluent B was acetonitrile (Avantor, Radnor, PA, USA) containing 0.1% formic acid. The following gradient elution program was used: 0 min, 5% B, 0–2 min, 5% B; 2–14 min, 100% B; 14–15 min, 100% B; 15–16 min, 5% B, 16–24 min, 5% B. A 1 μL aliquot of the samples was injected in every run. Samples were injected in a randomized order, after process blanks. The Q Exactive hybrid quadrupole-Orbitrap mass spectrometer (Thermo Fisher Scientific Inc., Waltham, MA, USA) was operated in polarity-switching mode. The capillary temperature was 320 °C, the voltage was 3.8 kV, and the gas flow was 32 (main and aux, respectively). The mass spectrometer was operated at 35,000 mass resolution, with an *m*/*z* range of 100–1500.

### 4.9. Examination of the Antioxidant Properties of Different Formulations

#### 4.9.1. DPPH Radical-Scavenging Assay

DPPH radical-scavenging activity was assessed using a modified method based on the work of Lee et al. [31]. In brief, 10 µL of the sample was mixed with 190 µL of DPPH solution (60 µM) in ethanol and incubated for 30 min in the dark at RT in a 96-well plate. Absorbance was measured at 517 nm using a microplate reader (Thermo Scientific™ Multiskan™ GO Microplate Spectrophotometer, Waltham, MA, USA). The percentage of DPPH radical-scavenging activity was calculated using the following equation:

(2) $$Scavenging\;activity\left(SA\%\right)=\left(\frac{A_{control}-A_{sample}}{A_{control}}\right)\times 100$$

where A_control_ is the absorbance of the DPPH solution without a sample and A_sample_ is the absorbance with the test sample.

#### 4.9.2. FRAP (Ferric Reducing Antioxidant Potential)

The FRAP assay is a commonly used method for measuring a sample’s antioxidant capacity based on its ability to reduce ferric ions (Fe^3+^) to ferrous ions (Fe^2+^). In this method, a ferric tripyridyltriazine (Fe^3+^-TPTZ) complex is reduced to a blue-colored ferrous form (Fe^2+^-TPTZ) in the presence of antioxidants. The intensity of the blue color, which is proportional to the antioxidant activity, is measured spectrophotometrically at 590 nm (Thermo Scientific™ Multiskan™ GO Microplate Spectrophotometer, USA). The FRAP assay kit (Sigma-MAK509, Sigma-Aldrich, St. Louis, MI, USA) was used to test the antioxidant effect, and the assay was performed according to the manufacturer’s instructions. Antioxidant activity was quantified in terms of ascorbic acid equivalents, based on a calibration curve generated from serial dilutions of ascorbic acid [83].

#### 4.9.3. CUPRAC (Cupric Reducing Antioxidant Capacity)

The antioxidant capacity of the samples was determined using the CUPRAC (cupric reducing antioxidant capacity) method, which is based on the reduction of Cu(II) to Cu(I) in the presence of neocuproine (2,9-dimethyl-1,10-phenanthroline) as a chromogenic ligand. In a 96-well plate, each well contained 50 µL of 10 mM CuCl_2_, 50 µL of 7.5 mM neocuproine (in 96% ethanol), 50 µL of 1 M ammonium acetate buffer (pH 7), 50 µL of distilled water, and 5 µL of the test sample. Absorbance was measured at 450 nm after incubation at RT for 30 min (Thermo Scientific™ Multiskan™ GO Microplate Spectrophotometer, USA). Antioxidant activity was expressed as ascorbic acid equivalents, using a standard calibration curve prepared from serial dilutions of ascorbic acid [84].

For all antioxidant assays, the results were normalized to appropriate controls and expressed as relative percentages. In the FRAP and CUPRAC assays, antioxidant activity was expressed relative to ascorbic acid, which was defined as 100%. In the DPPH assay, radical-scavenging activity was calculated relative to the DPPH control solution without sample. PBS was used as the negative control and background reference in all assays. All antioxidant assays were performed using equal volumes of formulations prepared with identical AA extract concentrations; therefore, comparisons reflect formulation-dependent modulation of AA-derived antioxidant activity, rather than differences in extract dose.

### 4.10. Cell Culture

The HaCaT (human immortalized non-tumorigenic keratinocyte) cell line was obtained from Cell Lines Service (CLS, Heidelberg, Germany). The culture medium was Dulbecco’s Modified Eagle’s Medium (DMEM) (Sigma Aldrich, Budapest, Hungary) supplemented with 1200 mM L-glutamine (Glutamax, Thermo-Fisher, Darmstadt, Germany, CAS: 35050061), 10% fetal bovine serum (FBS), 4.5 g/L glucose, and penicillin–streptomycin (0.1 mg/mL) (Sigma-Aldrich, Budapest, Hungary). Cells were incubated at 37 °C in 5% CO_2_ humidified air and maintained by continuous passaging. In experiments with HaCaT cells, the cells were passaged between passages 20 and 30.

### 4.11. MTT Assay

For cytotoxicity testing of the formulations, the human keratinocyte cell line (HaCaT) was cultured weekly. Cells were seeded at a 10,000 cells/well density on 96-well plates to achieve complete confluence. After draining the medium, the cells were washed with PBS and incubated with the test solutions for 2 h and 24 h at 37 °C in 5% CO_2_. After removing the test solutions, MTT solution (5 mg/mL) was added to the cells and incubated for 3 h in a light-free chamber. In the presence of living cells, the yellow tetrazolium dye was converted into purple formazan crystals by the action of oxidoreductase enzymes. The purple crystals were dissolved in a 25:1 ratio of isopropanol/hydrochloric acid. The absorbance of the samples was measured by a Thermo Scientific™ Multiskan™ GO Microplate Spectrophotometer (USA).

### 4.12. In Vitro Wound-Healing Assay

In vitro wound healing was assessed using the ibidi^®^ Culture-Insert 2 Well in µ-Dish 24 Well (Cat. No: 80242, ibidi GmbH, Gräfelfing, Germany) which creates a defined cell-free gap to evaluate cell migration. A cell suspension of 5 × 10^5^ cells was seeded per well and treated with AA extract at a concentration of 250 µg/mL, with or without excipients, diluted in standard culture medium. The assay was conducted following the manufacturer’s protocol. No proliferation inhibitor was applied during the assay. Wound closure was monitored microscopically to determine the effects of the treatments on cell migration and wound-healing capacity [85].

### 4.13. Experimental Animals

Rats have a larger body surface area than mice, enabling the generation of more standardized and readily quantifiable wounds, which is particularly advantageous for the evaluation and monitoring of topical formulations [86]. Sprague-Dawley and Wistar rats are commonly considered to be the most appropriate models for general wound-healing studies due to their ease of handling, well-characterized healing processes, and cost-effectiveness [87]. A total of 18 male Sprague-Dawley albino rats (250–350 g) were used for the wound-healing study. The animals were provided by AnimaLab Hungary Kft (Vác, Hungary). The animals were kept in the experimental environment for one week to acclimatize them to the experimental conditions in the Animal House of the Department of Pharmacology, Faculty of Pharmacy, University of Debrecen (registration number: III/12-KÁT/2024). The day before surgery, the animals were given intramuscular gentamicin injections (5 mg/kg body weight) to avoid infection. The gentamicin dose was adjusted according to the *United States Pharmacopeia* (USP) gentamicin sulfate solution for injection administration in rats. Animals were housed on a 12 h L/D cycle in autoclavable PC cages (904 cm^2^ floor area), with 2–3 animals per cage. Animals were kept optimally at 22 ± 2 °C, with a relative humidity 55 ± 10%, and with continuous mechanical air exchange with filtered air circulation. The stocking density was set according to the European Commission Recommendation 2007/526/EC, Directive 2010/63/EU.

The animals had not been subjected to any previous experimental interventions. Upon completion of the study, euthanasia was carried out via administration of ip. pentobarbital sodium (100 mg/kg). No animals or data points were excluded from the analysis. No inclusion or exclusion criteria were specified in advance.

### 4.14. In Vivo Wound-Healing Assay

**Figure 9:**
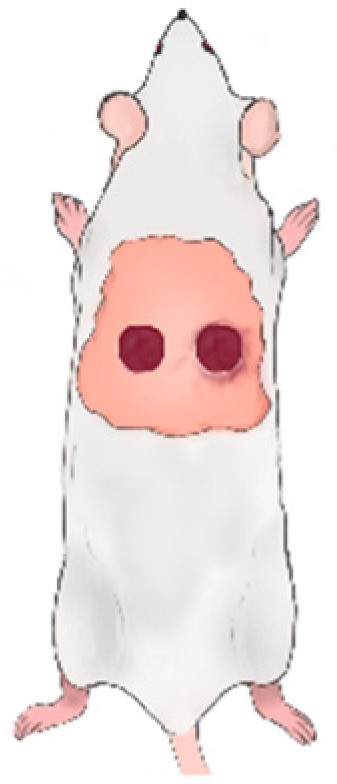
The location of the rats’ wounds is in the dorsal region.

During the study, four experimental groups were established: an untreated control group, a group treated with empty hydrogel, a group treated with hydrogel V., and a group treated with hydrogel VIII. Animals were randomly assigned to treatment groups using a simple randomization procedure. Investigators responsible for wound measurements and data analysis were aware of the treatment allocation. A total of 18 animals were included in the experiment, which was performed on three independent occasions with six animals per session, resulting in two animals per experimental group in each session. The animals were anesthetized on the day of surgery by an injection of ketamine/xylazine (50/10 mg/kg body weight) and, after anesthesia was complete, two regular circular wounds (d = 1.25 cm) were made in the hairless back of the animal using a sterile surgical stainless steel scalpel, scissors, and forceps. The dermis, epidermis, and subcutaneous layers were removed during surgery. The wound margins were smeared with a 10 mg/mL free active iodine disinfectant solution, photographed, and the hydrogel formulations to be examined were placed on the wound. The left wounds of the animals were dressed with a drug-free hydrogel or left blank (untreated control), as shown in Figure 9. For the right wound, animals were dressed with a hydrogel containing AA or a hydrogel containing AA and excipients. The wounds were sealed with sterile dressings (adhesive plasters, gauze pads).

To analyze the wound-healing process, the hydrogels were replaced every 24 h for 18 days, and a new dressing was applied to the rats. Any contamination of the wounds was washed with sterile physiological saline. Rats were anesthetized with a 2–4% isoflurane anesthetic machine to allow for dressing changes and recording (R500—Compact Small Animal Anesthesia Machine, RWD-China, Shenzhen, China)). Sample size was determined based on previous wound-healing studies using similar rat excision models, balancing statistical robustness with the principles of the 3Rs. No formal a priori power calculation was performed [88]. No histological or immunohistochemical analyses were performed.

Progressive changes in wound healing were quantitatively evaluated using ImageJ 1.52 software. Wound area was calculated from scaled images of the wounds taken on days 0, 3, 5, 7, 10, and 12. The percentage of healing was calculated using the following equation, where *n* = number of days (3, 5, 7, 10, or 12) for which the area had healed:

(3) $$Wound\;healing\;percentage\left(\%\right)=\frac{\left(wound\;area\;(day\;0)\right)-\left(wound\;area\;(day\;n)\right)}{\left(wound\;area\;(day\;0)\right)}\times 100$$

### 4.15. Statistical Analysis

The results of the experiment were analyzed using Microsoft Office Excel 2016, and the data were graphically and statistically analyzed using GraphPad Prism (version 8, GraphPad Software, San Diego, CA, USA). The results presented in this paper are expressed as the mean ± SEM. All data were obtained from a minimum of three independent experiments, and at least three parallel concentrations were used within each experiment. Raw results were compared by one-way ANOVA and repeated-measures ANOVA followed by Tukey or Dunnett post-testing. Statistical significance for the difference of means was assigned into one of five categories: *p* > 0.05 (not significant), *p* < 0.05 (*), *p* < 0.01 (**), *p* < 0.001 (***), or *p* < 0.0001 (****). In the in vivo wound-healing experiment, two wounds were generated per animal. Each animal therefore contributed paired measurements. Statistical analyses were primarily performed at the wound level. Although this approach allows for comparisons between treatment groups, it does not fully account for potential within-animal dependence. Accordingly, statistical inferences from in vivo data should be interpreted cautiously, and future studies should apply animal-level aggregation or mixed-effects models to provide more robust estimates.

## 5. Conclusions

This study systematically evaluated *Artemisia annua*-loaded alginate hydrogels formulated with hyaluronic acid and dexpanthenol, demonstrating that the incorporation of these excipients enhances hydration stability, antioxidant performance, and biological activity. All AA-containing hydrogels exhibited biocompatibility and promoted wound closure, with the combined HA–DP formulation producing the most pronounced in vitro and in vivo effects. These findings confirm that synergistic hydrogel systems can substantially improve the therapeutic potential of AA for topical wound management and support further development toward clinical or cosmeceutical applications. Future studies employing systematic excipient-only control formulations and factorial experimental designs are warranted to further elucidate the individual and interactive roles of *Artemisia annua*, hyaluronic acid, and dexpanthenol.

## Figures and Tables

**Table 1:** Moisture content (MC%), water-retention capacity (WRR%), pH, and gelation time (GT) values of the different *AA*-supplemented hydrogels (V.–VIII.).

Entry	MC%	WRR%	pH	GT (s)
V.	97.04 ± 1.36	74.12 ± 2.19	5.39 ± 0.15	234 ± 1.1
VI.	96.69 ± 1.97	78.24 ± 1.63	5.15 ± 0.23	212 ± 1.5
VII.	90.70 ± 3.66	72.25 ± 2.18	5.28 ± 0.17	198 ± 0.8
VIII.	88.90 ± 2.14	77.53 ± 2.94	5.32 ± 0.21	182 ± 1.2

Values are expressed as the mean ± S.D., *n* = 5.

**Table 2:** Comparison of the different preparations shown in Figure 3. We performed a simple one-way ANOVA with Dunnett’s multiple comparison test. Adjustment of *p*-values was performed using the Tukey–Kramer method; **** indicates statistically significant differences at *p* < 0.0001.

Dunnett’s Multiple Comparison Test	Mean Diff.	95.00% CI of Diff.	Below Threshold?	Summary	Adjusted *p*-Value
PBS vs. Asc. acid 0.5%	−95.29	−95.53 to −95.06	Yes	****	<0.0001
PBS vs. V.	−56.61	−56.65 to −56.58	Yes	****	<0.0001
PBS vs. VI.	−68.7	−68.80 to −68.61	Yes	****	<0.0001
PBS vs. VII.	−61.52	−61.57 to −61.48	Yes	****	<0.0001
PBS vs. VIII.	−71.82	−71.94 to −71.69	Yes	****	<0.0001

**Table 3:** Comparison of the different preparations shown in Figure 4. We performed a simple one-way ANOVA with Dunnett’s multiple comparison test. Adjustment of *p*-values was performed using the Tukey–Kramer method; **** indicates statistically significant differences at *p* < 0.0001.

Dunnett’s Multiple Comparison Test	Mean Diff.	95.00% CI of Diff.	Below Threshold?	Summary	Adjusted *p*-Value
PBS vs. DPPH stock sol.	−95.78	−98.80 to −92.75	Yes	****	<0.0001
PBS vs. Asc. acid 0.5%	−91.31	−92.01 to −90.61	Yes	****	<0.0001
PBS vs. V.	−60.18	−60.64 to −59.71	Yes	****	<0.0001
PBS vs. VI.	−69.23	−69.39 to −69.07	Yes	****	<0.0001
PBS vs. VII.	−62.18	−62.30 to −62,05	Yes	****	<0.0001
PBS vs. VIII.	−72.62	−72.79 to −72.45	Yes	****	<0.0001

**Table 4:** Comparison of the different preparations shown in Figure 5. We performed a simple one-way ANOVA with Dunnett’s multiple comparison test; *p*-value adjustments were performed using the Tukey–Kramer method; **** indicates statistically significant differences at *p* < 0.0001.

Dunnett’s Multiple Comparison Test	Mean Diff.	95.00% CI of Diff.	Below Threshold?	Summary	Adjusted *p*-Value
PBS vs. Asc. acid 0.5%	−95.79	−95.92 to −95.66	Yes	****	<0.0001
PBS vs. V.	−32	−32.06 to −31.94	Yes	****	<0.0001
PBS vs. VI.	−35.38	−35.52 to −35.24	Yes	****	<0.0001
PBS vs. VII.	−34.67	−34.75 to −34.58	Yes	****	<0.0001
PBS vs. VIII.	−35.92	−36.03 to −35.81	Yes	****	<0.0001

**Table 5:** To compare the different preparations shown in Figure 6 with PBS, we performed a simple one-way ANOVA with Dunnett’s multiple comparison test. Adjustment of *p*-values was performed using the Tukey–Kramer method; and statistical significance is indicated as follows: ns, not significant (*p* ≥ 0.05); * *p* < 0.05; ** *p* < 0.01; *** *p* < 0.001; **** *p* < 0.0001.

Tukey’s Multiple Comparisons Test	Significant?	Summary	Adjusted *p*-Value
DMSO vs. Control	Yes	**	0.0098
DMSO vs. VIII. 100 μg	No	ns	0.5905
DMSO vs. VIII. 250 μg	No	ns	0.9833
DMSO vs. VI. 100 μg	Yes	**	0.0057
DMSO vs. VI. 250 μg	Yes	****	<0.0001
DMSO vs. VII. 100 μg	Yes	***	0.0001
DMSO vs. VII. 250 μg	No	ns	0.2702
DMSO vs. V. 100 μg	No	ns	0.5526
DMSO vs. V. 250 μg	No	ns	0.9674
Control vs. VIII. 100 μg	Yes	****	<0.0001
Control vs. VIII. 250 μg	No	ns	0.1617
Control vs. VI. 100 μg	No	ns	>0.9999
Control vs. VI. 250 μg	No	ns	0.1433
Control vs. VII. 100 μg	Yes	****	<0.0001
Control vs. VII. 250 μg	No	ns	0.9351
Control vs. V. 100 μg	Yes	****	<0.0001
Control vs. V. 250 μg	Yes	***	0.0002
VIII. 100 μg vs. VIII. 250 μg	No	ns	0.0818
VIII. 100 μg vs. VI. 100 μg	Yes	****	<0.0001
VIII. 100 μg vs. VI. 250 μg	Yes	****	<0.0001
VIII. 100 μg vs. VII. 100 μg	No	ns	0.0667
VIII. 100 μg vs. VII. 250 μg	Yes	**	0.0013
VIII. 100 μg vs. V. 100 μg	No	ns	>0.9999
VIII. 100 μg vs. V. 250 μg	No	ns	0.9981
VIII. 250 μg vs. VI. 100 μg	No	ns	0.1086
VIII. 250 μg vs. VI. 250 μg	Yes	****	<0.0001
VIII. 250 μg vs. VII. 100 μg	Yes	****	<0.0001
VIII. 250 μg vs. VII. 250 μg	No	ns	0.9033
VIII. 250 μg vs. V. 100 μg	No	ns	0.0714
VIII. 250 μg vs. V. 250 μg	No	ns	0.4057
VI. 100 μg vs. VI. 250 μg	No	ns	0.2086
VI. 100 μg vs. VII. 100 μg	Yes	****	<0.0001
VI. 100 μg vs. VII. 250 μg	No	ns	0.8709
VI. 100 μg vs. V. 100 μg	Yes	****	<0.0001
VI. 100 μg vs. V. 250 μg	Yes	***	0.0001
VI. 250 μg vs. VII. 100 μg	Yes	****	<0.0001
VI. 250 μg vs. VII. 250 μg	Yes	**	0.0037
VI. 250 μg vs. V. 100 μg	Yes	****	<0.0001
VI. 250 μg vs. V. 250 μg	Yes	****	<0.0001
VII. 100 μg vs. VII. 250 μg	Yes	****	<0.0001
VII. 100 μg vs. V. 100 μg	No	ns	0.0766
VII. 100 μg vs. V. 250 μg	Yes	**	0.0072
VII. 250 μg vs. V. 100 μg	Yes	**	0.001
VII. 250 μg vs. V. 250 μg	Yes	*	0.0149
V. 100 μg vs. V. 250 μg	No	ns	0.9969

**Table 6:** (**A**) Linear regression and (**B**) two-way ANOVA with Geisser–Greenhouse correction analysis of wound closure in vivo. The table presents the slope, intercept, confidence intervals, and goodness-of-fit parameters for untreated wounds and those treated with formulations I, V, and VIII. **** indicates statistically significant differences at *p* < 0.0001.

(**A**)					
**Simple Linear Regression**	**Untreated**	**I.**		**V.**	**VIII.**
Best-fit values					
Slope	−7.358	−7.579		−7.851	−7.948
Y-intercept	105.6	105		97.83	96.85
X-intercept	14.36	13.85		12.46	12.19
1/slope	−0.1359	−0.1319		−0.1274	−0.1258
Std. Error					
Slope	0.8514	0.8098		0.5494	0.7161
Y-intercept	6.05	5.755		3.904	5.089
95% Confidence Intervals					
Slope	−9.722 to −4.994	−9.828 to −5.331		−9.376 to −6.325	−9.936 to −5.960
Y-intercept	88.85 to 122.4	89.02 to 121.0		86.99 to 108.7	82.72 to 111.0
X-intercept	11.99 to 18.69	11.73 to 17.52		11.16 to 14.28	10.62 to 14.60
Goodness of Fit					
R squared	0.9492	0.9563		0.9808	0.9686
Sy.x	8.464	8.051		5.462	7.119
Is slope significantly non-zero?					
F	74.69	87.6		204.2	123.2
DFn, DFd	1, 4	1, 4		1, 4	1, 4
*p*-value	0.001	0.0007		0.0001	0.0004
Deviation from zero?	Significant	Significant		Significant	Significant
Equation	Y = −7.358 × X + 105.6	Y = −7.579 × X + 105.0		Y = −7.851 × X + 97.83	Y = −7.948 × X + 96.85
(**B**)					
**Two-Way RM ANOVA**	**Matching: Stacked**				
Assume sphericity?	No				
Alpha	0.05				
**Source of Variation**	**% of Total Variation**	***p*** **-Value**	***p*** **-Value Summary**	**Significant?**	**Geisser-Greenhouse’s Epsilon**
Row Factor x Column Factor	1.399	<0.0001	****	Yes	
Row Factor	92.88	<0.0001	****	Yes	0.6207
Column Factor	3.019	<0.0001	****	Yes	
Subject	0.4884	0.3593	ns	No	
ANOVA Table	SS	DF	MS	F (DFn, DFd)	*p*-Value
Row Factor x Column Factor	2145	15	143	F (15, 100) = 4.212	*p* < 0.0001
Row Factor	142,337	5	28,467	F (3.103, 62.07) = 838.6	*p* < 0.0001
Column Factor	4626	3	1542	F (3, 20) = 41.21	*p* < 0.0001
Subject	748.5	20	37.42	F (20, 100) = 1.102	*p* = 0.3593
Residual	3395	100	33.95		

**Table 7:** The compositions of the different gel formulations.

Entry	SA	CaCO_3_	GDL	HA	DP	AA
I.	+	+	+	−	−	−
II.	+	+	+	+	−	−
III.	+	+	+	−	+	−
IV.	+	+	+	+	+	−
V.	+	+	+	−	−	+
VI.	+	+	+	+	−	+
VII.	+	+	+	−	+	+
VIII.	+	+	+	+	+	+

## Data Availability

The original contributions presented in this study are included in the article and Supplementary Material. Further inquiries can be directed to the corresponding author.

## Supplementary Materials

The following supporting information can be downloaded at https://www.mdpi.com/article/10.3390/ph19030424/s1, Figure S1: Representative EICs (extracted ion chromatograms) of the LC-MS detection of casticin from the acceptor phase, showing alignment of the calibration curves from an authentic standard with the peaks detected from the plant extract. Figure S2: Representative EICs (extracted ion chromatograms) of the LC-MS detection of casticin from the EtOH extract of the used plant material, showing alignment of the calibration curves from an authentic standard with the peaks detected from the plant extract. Figure S3: Representative EICs (extracted ion chromatograms) of the LC-MS detection of arteannuin B from the EtOH extract of the used plant material, showing alignment of the calibration curves from an authentic standard with the peaks detected from the plant extract. Figure S4: Representative EICs (extracted ion chromatograms) of the LC-MS detection of artemetin from the EtOH extract of the used plant material, showing alignment of the calibration curves from an authentic standard with the peaks detected from the plant extract. Figure S5: Representative EICs (extracted ion chromatograms) of the LC-MS detection of deoxyartemisinin from the EtOH extract of the used plant material, showing alignment of the calibration curves from an authentic standard with the peaks detected from the plant extract. Figure S6: Representative EICs (extracted ion chromatograms) of the LC-MS detection of dihydroartemisinic acid from the EtOH extract of the used plant material, showing alignment of the calibration curves from an authentic standard with the peaks detected from the plant extract. Table S1: Analytical performance of the chosen method, covering the relevant range of determination for each bioactive constituent of *Artemisia annua*. All data are given for measurements in positive ion mode in the range LLOQ-ULOQ in MS^1^. The ULOQ was 20 µg/mL for all metabolites. Table S2: Additional MS data acquisition parameters.
