# Supplementary material for: Preparation and Investigation of Artemisia annua L.-Loaded Alginate Hydrogels with Excipients
Source: Pharmaceuticals (Basel). 2026 Mar 5;19(3):424. doi: 10.3390/ph19030424 (PMC13028784; doi:10.3390/ph19030424)
Supplement: Supplementary file 1 [file pharmaceuticals-19-00424-s001.zip › pharmaceuticals-4139755-supplementary.pdf]

Supplementary Materials

Preparation and investigation of *Artemisia annua* L. loaded alginate hydrogels with excipients

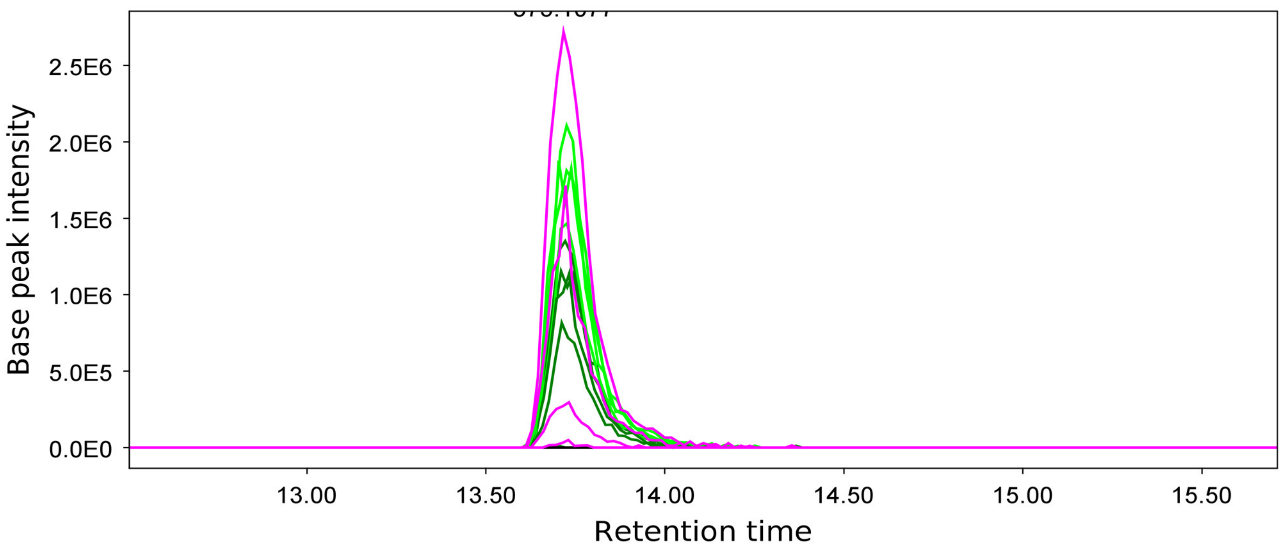

Figure S1. Representative EICs (extracted ion chromatograms) of the LC-MS detection of casticin from the acceptor phase showing alignment of the calibration curves from an authentic standard with the peaks detected from the plant extract.

Chromatograms were exported from raw data with mzMine 4.8.30, the m/z range shown is 375.1077±5 ppm in position ion mode. Casticin: $C_{19}H_{19}O_8^+$; calcd., 375.10799; meas., 375.1077, delta = +0.77 ppm. Color scheme: black, blanks at 0h and 5h (unseen as covered by calibration curve baseline); dark green, acceptor phases at 3h; light green, acceptor phases at 5h; magenta, relevant subset of calibration curve points (0.001, 0.01, 0.05 and 0.1 µg mL^{-1}).

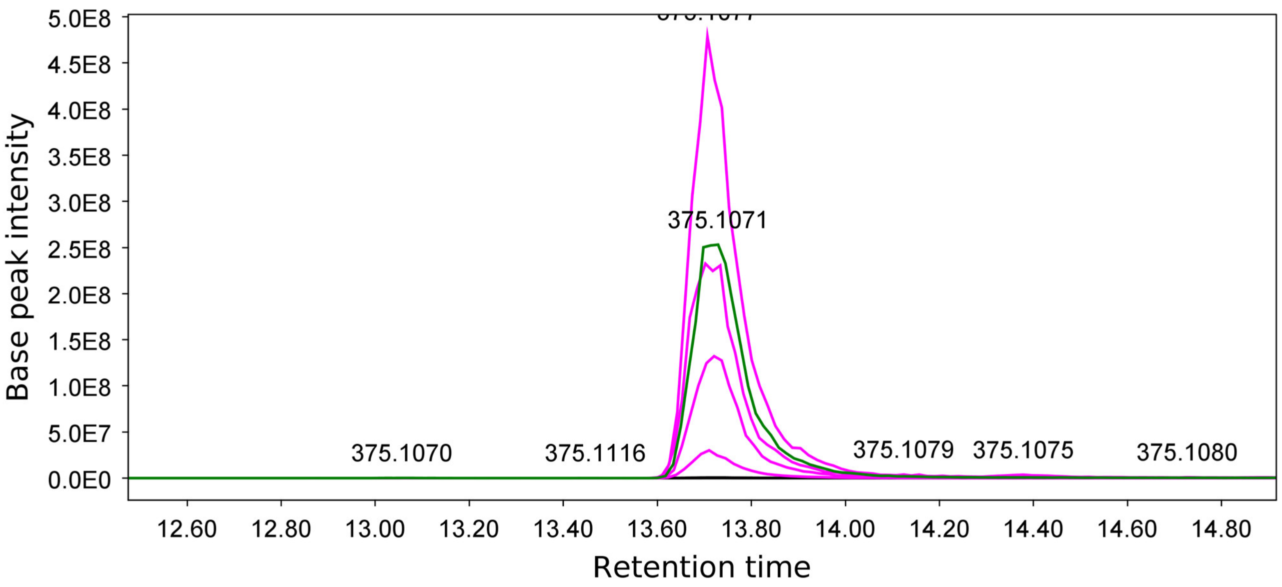

Figure S2. Representative EICs (extracted ion chromatograms) of the LC-MS detection of casticin from the EtOH extract of the used plant material showing alignment of the calibration curves from an authentic standard with the peaks detected from the plant extract.

Chromatograms were exported from raw data with mzMine 4.8.30, the m/z range shown is 375.1077±5 ppm in position ion mode. Casticin: $C_{19}H_{19}O_8^+$; calcd., 375.10799; meas., 375.1077, delta = +0.77 ppm. Color scheme: black, process blank; dark green, EtOH extract, injection was 100 µg DW equivalent; magenta, relevant subset of calibration curve points (0.5, 1, 5, 10, 20 µg mL^{-1}).

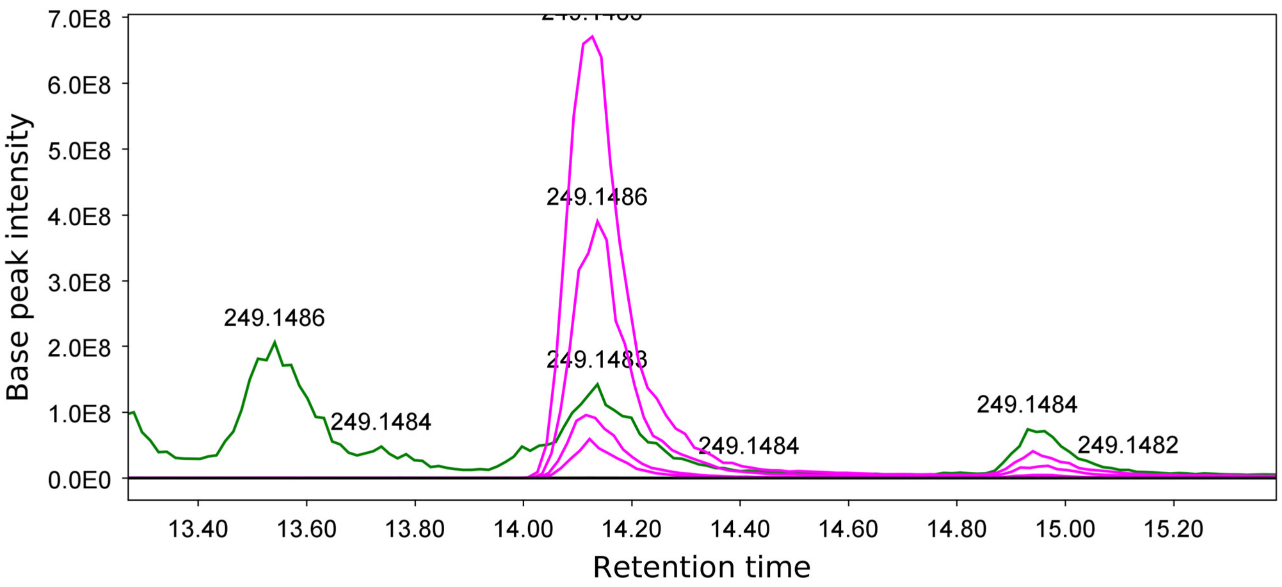

Figure S3. Representative EICs (extracted ion chromatograms) of the LC-MS detection of arteannuin B from the EtOH extract of the used plant material showing alignment of the calibration curves from an authentic standard with the peaks detected from the plant extract.

Chromatograms were exported from raw data with mzMine 4.8.30, the m/z range shown is 249.1486±5 ppm in position ion mode. Arteannuin B: $C_{15}H_{21}O_3^+$; calcd., 249.14907; meas., 249.1486, delta = -1.88 ppm. Color scheme: black, process blank; dark green, EtOH extract, injection was 100 µg DW equivalent; magenta, relevant subset of calibration curve points (0.05, 0.1, 0.5, 1 µg mL^{-1}).

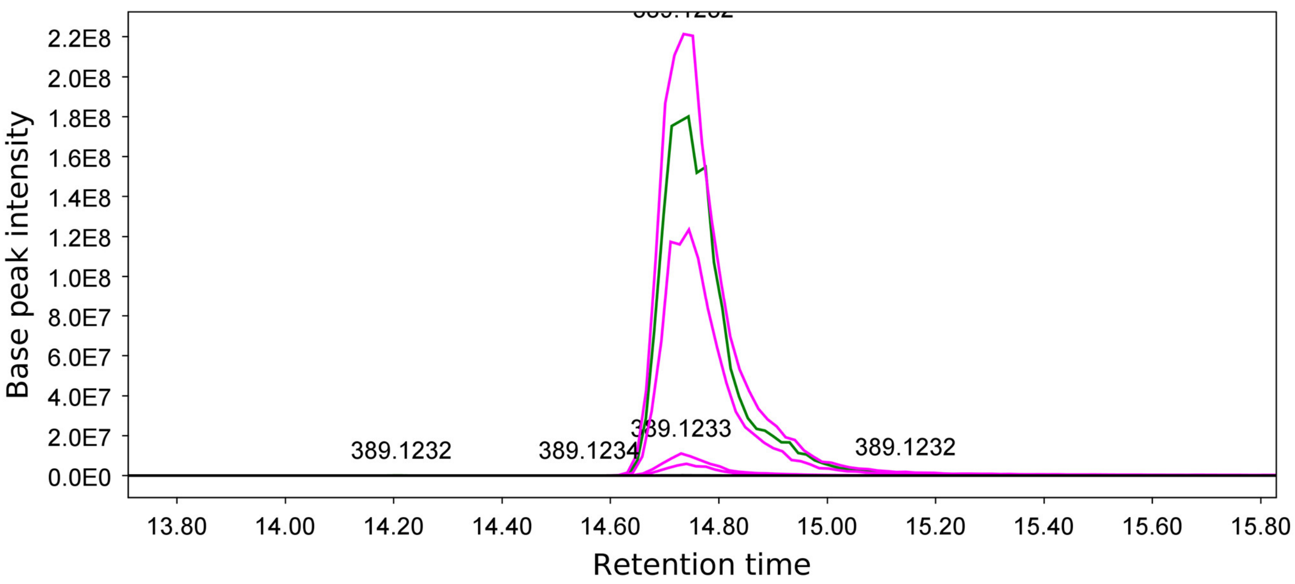

Figure S4. Representative EICs (extracted ion chromatograms) of the LC-MS detection of artemetin from the EtOH extract of the used plant material showing alignment of the calibration curves from an authentic standard with the peaks detected from the plant extract.

Chromatograms were exported from raw data with mzMine 4.8.30, the m/z range shown is 389.1233±5 ppm in position ion mode. Artemetin: $C_{20}H_{21}O_8^+$; calcd., 389.1236; meas., 389.1233, delta = 0.77 ppm. Color scheme: black, process blank; dark green, injection was 1 mg DW equivalent; magenta, relevant subset of calibration curve points (0.1, 0.5, 1, 5, 10 μg mL^{-1}).

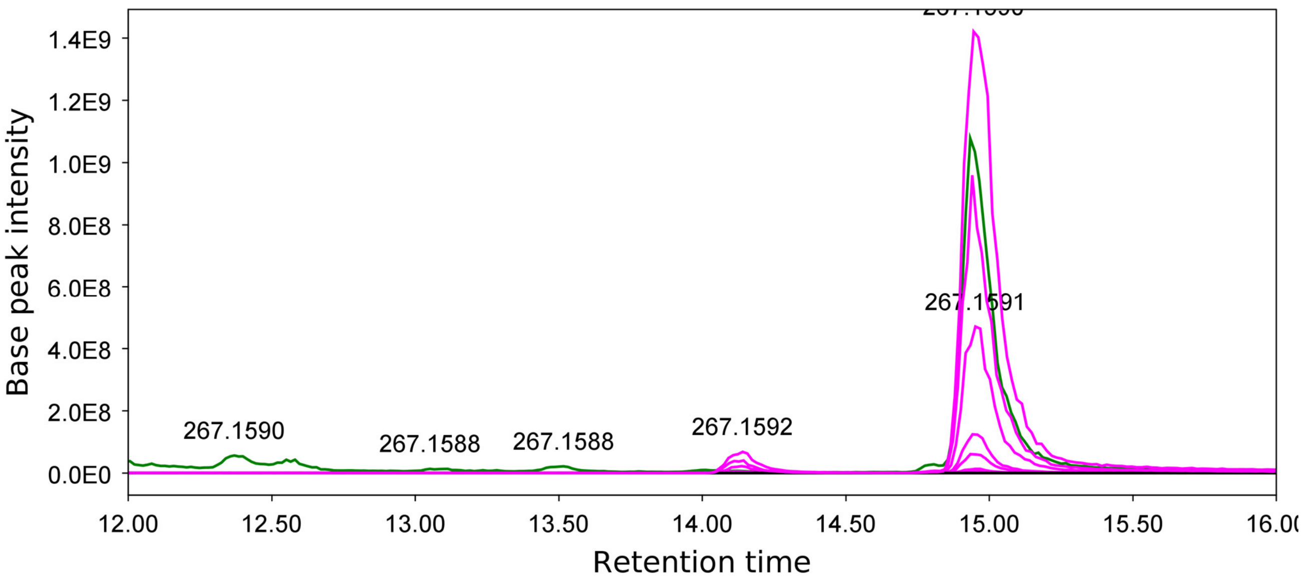

Figure S5. Representative EICs (extracted ion chromatograms) of the LC-MS detection of deoxyartemisinin from the EtOH extract of the used plant material showing alignment of the calibration curves from an authentic standard with the peaks detected from the plant extract.

Chromatograms were exported from raw data with mzMine 4.8.30, the m/z range shown is 267.1591±5 ppm in position ion mode. Deoxyartemisinin: $C_{15}H_{23}O_4^+$; calcd., 267.1596; meas., 267.1591, delta = +1.98 ppm. Color scheme: black, process blank (unseen as covered by baseline); dark green, injection was 500 µg DW equivalent; magenta, relevant subset of calibration curve points (0.1, 0.5, 1, 5, 10, 20 µg mL^{-1}).

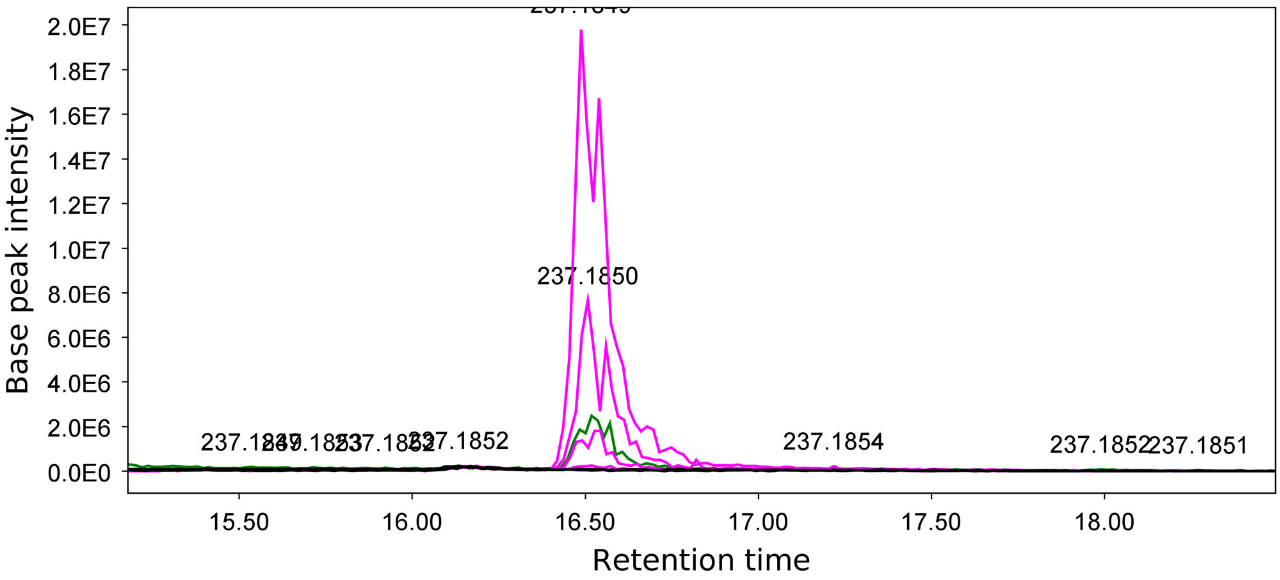

Figure S6. Representative EICs (extracted ion chromatograms) of the LC-MS detection of dihydroartemisinic acid from the EtOH extract of the used plant material showing alignment of the calibration curves from an authentic standard with the peaks detected from the plant extract.

Chromatograms were exported from raw data with mzMine 4.8.30, the m/z range shown is 237.1850±5 ppm in position ion mode. Dihydroartemisinic acid: $C_{15}H_{25}O_2^+$; calcd., 237.1855; meas., 237.1850, delta = +2.11 ppm. Color scheme: black, process blank (unseen as covered by baseline); dark green, injection was 200 µg DW equivalent; magenta, relevant subset of calibration curve points (0.1, 0.5, 5, 20 µg mL^{-1}).

Table S1. Analytical performance of the chosen method covering the relevant range of determination for each bioactive constituent of *Artemisia annua*. All data are given for measurements in positive ion mode in the range LLOQ - ULOQ in MS^1. ULOQ was 20 µg/mL for all metabolites.

**Metabolite**	**R^2 [a]**	**Linear equation**	**LLOQ[b],µg/mL**	**In extract[c], µg/mL**
Arteannuin B	0.9940	y = 7.90E6 x + 2.53E6	0.01	0.23
Artemetin	0.9982	y = 2.98E6 x - 2.68E6	0.05	1.74
Casticin	0.9980	y = 2.89E6 x + 5.02E6	0.05	11.21
Deoxyartemisinin	0.9964	y = 1.07E7 x + 2.84E6	0.05	2.46
Dihydroartemisinic acid	0.9878	y = 1.14E5 x + 8.02E4	0.5	1.17
Artemisinin	0.9950	y = 2.12E6 x + 5.52E5	0.1	<LLOQ

Notes: [a], linearity of the calibration curve; [b], lower limit of quantitation, the lowest point of the calibration curve where signal height is greater than 10-fold the height of background noise; [c], In-solution concentration of analytes in the 100 mg DW/mL EtOH extract.

Table S2. Additional MS data acquisition parameters.

Capillary temperature	320 °C
Voltage	3.8 kV
Gas flow (arb)	32
Aux glas flow (arb)	7
Polarity switching frequency	Approx. 1Hz
Resolution	35,000
m/z range	100-1500
